# Bacterial Acute Otitis Media Complicated with Otorrhea in a Children’s Hospital in the Era of Pneumococcal Conjugate Vaccines

**DOI:** 10.3390/pathogens14050494

**Published:** 2025-05-17

**Authors:** Irene Tzovara, Anastasios Doudoulakakis, Georgios Kalogeras, Emmanouil Koutouzis, Charilaos Dellis, Sophia Pasparakis, Marietta Charakida, Evangelia Lebessi, Elisavet Bozavoutoglou, Michael Tsakanikos, Vassiliki Syriopoulou, Maria Tsolia

**Affiliations:** 1 First Department of Pediatrics, School of Medicine, National and Kapodistrian University of Athens, “Aghia Sophia” Children’s Hospital, 11527 Athens, Greece; itzovara@med.uoa.gr; 2 Department of Microbiology, “P. & A. Kyriakou” Children’s Hospital, 11527 Athens, Greece; 3 Infectious Diseases and Chemotherapy Research Laboratory, First Department of Pediatrics, School of Medicine, National and Kapodistrian University of Athens, “Aghia Sophia” Children’s Hospital, 11527 Athens, Greece; 4 Second Department of Pediatrics, School of Medicine, National and Kapodistrian University of Athens, “P. & A. Kyriakou” Children’s Hospital, 11527 Athens, Greece; 5 Ear Nose and Throat Department, “P. & A. Kyriakou” Children’s Hospital, 11527 Athens, Greece

**Keywords:** acute otitis media, otorrhea, pneumococcal conjugate vaccine, pneumococcal serotypes, surveillance, antibiotic resistance

## Abstract

Acute otitis media (AOM) is a common disease among children and can be complicated by otorrhea (AOMO). In 2010, the 13-valent Pneumococcal Conjugate Vaccine (PCV13) replaced the 7-valent vaccine (PCV7) in Greece. We aimed to describe the microbiological profile of bacterial ΑOMO among children younger than 16 years across the two PCV periods in a tertiary children’s hospital. Middle ear fluid cultures from 2418 children with AOMO were collected from 2007 to 2022. Otopathogens were isolated and tested for antimicrobial susceptibility. Data were compared between the PCV7- (2007–2011) and PCV13-period (2012–2019). The most common otopathogen over the 16-year period was *S. pyogenes* (35.4%), followed by *H. influenzae* (33.8%), *S. pneumoniae* (26.6%), and *M. catarrhalis* (4.1%). Pneumococcal resistance to cefotaxime and clindamycin significantly increased from 2% to 4.5% (*p* = 0.019) and 16.1% to 22.8% (*p* = 0.039), respectively. Resistance of *H. influenzae* to ampicillin increased from 6.3% to 13.9% (*p* < 0.001). A significant reduction in cotrimoxazole-resistant *S. pneumoniae* from 31% to 22.4% (*p* = 0.012), and in clindamycin-resistant and erythromycin-resistant *S pyogenes*, from 17.4% to 9.3% and 21.4% to 10.8%, respectively (*p* ≤ 0.001), was observed. During 2013–2022, 38 *S. pneumoniae* serotypes were identified among 250 isolates. Serotype 3 (27.2%) and 19A (13.2%) prevailed, followed by 19F (7%). The most common causes after the shift to PCV13 are *S. pyogenes* and *H. influenzae*. However, *S. pneumoniae* remains an important otopathogen with significant antimicrobial resistance. Serotype 3 was mostly detected, followed by 19A.

## 1. Introduction

Acute Otitis Media (AOM) represents one of the most common childhood infections, with increased incidence in children between 6 months and 2 years of age [1]. Before the 21st century, approximately 80% of children had at least one episode of AOM in the first 3 years of life, and almost 40% of them suffered from recurrent episodes during their childhood [2]. Though mild and asymptomatic in most cases, AOM poses a significant socioeconomic burden on families and national healthcare systems [3,4,5]. Even today, AOM represents a leading cause of outpatient and emergency department visits, keeping children from school/daycare and parents from work [6]. It is the most common pediatric infection [7,8] and one of the leading causes of antibiotic prescribing, as it is often over-diagnosed and treated. According to the American Academy of Pediatrics, more than 10,000,000 antibiotic prescriptions are attributed to AOM yearly [9,10]. In Greece, during 2010–2013, AOM was responsible for 22.3% of the total antibiotics prescribed to children and adolescents [11].

AOM can be complicated by otorrhea (AOM with Otorrhea, AOMO) in up to 15–10% of cases, either after tympanic membrane perforation or through ear tubes [12,13]. The same bacterial pathogens causing uncomplicated AOM, also called otopathogens, are responsible for AOMO. These include *Streptococcus pneumoniae* or pneumococcus, *Haemophilus influenzae*, *Streptococcus pyogenes*, and *Moraxella catarrhalis* [14].

Before the use of Pneumococcal Conjugate Vaccines (PCVs), almost 80% of AOM cases were attributed to *S. pneumoniae* and *H. influenzae*, with pneumococcus being responsible for 40–50% of those cases [15]. The implementation of universal infant immunization with PCVs since the early 2000s has led to a substantial decrease in pneumococcal AOM, as reported in the latest Cochrane Review in 2020 [16]. However, data regarding their impact on all-cause bacterial AOM have been conflicting, with some studies showing a decrease—albeit to a lesser degree [16,17,18,19,20].

In Greece, the 7-valent PCV (PCV7) vaccine was introduced in the National Immunization Program (NIP) in 2006 and was replaced by the 13-valent PCV (PCV13) in 2010. In the years following the introduction of PCV7, a 38% decrease in AOMO cases examined at a tertiary children’s hospital in Athens was observed, together with an emerging shift in the predominant bacteria from *S. pneumoniae* to *H. influenzae* [21]. However, regional data on AOMO in the era of PCV13 have been scarce. The aim of this study was to describe the microbiological profile of bacterial AOMO among children younger than 16 years of age in our setting across the two PCV periods, and to assess potential temporal trends in pathogen distribution.

## 2. Materials and Methods

This retrospective observational study was conducted at the “P. & A. Kyriakou” Children’s Hospital in Athens, one of the two tertiary children’s hospitals in Athens, serving a population of approximately 600,000 children younger than 16 years of age. This study was approved by the hospital’s Ethics Review Board.

All culture-positive AOMO cases during a 16-year period, from 1 January 2007 to 31 December 2022, were recorded. Culture-positive AOMO cases were defined as children with middle ear fluid (MEF) cultures positive for bacterial otopathogens. MEF samples were obtained mainly from spontaneously ruptured tympanic membranes, as tympanocentesis is only rarely performed at our setting, thus representing AOMO cases. Cultures from bilateral cases and successive AOMO episodes within a month were considered as a single episode. Demographic characteristics of the patients (age and gender) were available from the hospital medical records.

The study period was divided into two PCV-periods based on the shift from PCV7 to PCV13 in the NIP in 2010. The year 2011 was considered a transitional year and was included in the PCV7-period (2007–2011). The PCV13-period included the years 2012 to 2019. To avoid confounding the data regarding the impact of the shift to PCV13 on AOMO from changes due to the COVID-19 pandemic, the years 2020–2022 were excluded from the PCV13-period. However, data from 2020–2022 were included when reporting the demographic characteristics of the patients and serotyping of *S. pneumoniae* isolates.

### 2.1. Microbiology

MEF specimens were plated on Colombia blood agar with 5% sheep blood and on chocolate agar, then incubated at 35 °C in 5% CO_2_ for 24 to 48 hours. *S. pneumoniae* strains were identified by typical alpha hemolysis on blood agar, susceptibility to optochin, and the bile solubility test. *S. pyogenes* was identified by beta hemolysis on blood agar, susceptibility to bacitracin 0.04 units (Oxoid, Basingstoke Hants, UK), and serogrouping (Streptex, Remel Europe, Dartford, UK). *H. influenzae* was identified by its growth requirements for factors V and X (Oxoid, Basingstoke Hants, UK). *M. catarrhalis* was identified by VITEK NH (bioMerieux SA, Marcy-l’Etoile, France). Cultures were performed with standard methods and susceptibility was performed with the Disk Diffusion Method according to CLSI until 2017 and EUCAST recommendations after 2017. For pneumococcal strains tested with a ≤20 mm zone to oxacillin 1 μg disc, MICs to penicillin, amoxicillin or ampicillin, and cefotaxime were determined using a gradient method (ETEST^®^, bioMerieux, Marcy l’Etoile, France). Isolates were categorized according to the EUCAST updated definitions as either susceptible-standard dosing regimen (S), susceptible-increased exposure (I), or resistant (R). Susceptibility testing was performed simultaneously for all relevant antibiotics for each isolate using the standardized protocol in place at the time. Minor differences in the total number of isolates tested per antibiotic reflect occasional missing data entries in the electronic records and not selective testing or exclusion of specific antibiotics. A number of pneumococcal strains isolated from 2013 onwards were stored in skim milk at −70 °C. Pneumococcal serogrouping was performed using the latex agglutination method (Pneumotest-latex, Statens Serum Institute, Copenhagen, Denmark), and serotyping was carried out with the capsular swelling method using specific antisera (Pneumococcal factor antisera, Statens Serum Institute, Copenhagen, Denmark).

### 2.2. Statistical Analysis

Numerical data (e.g., age) were expressed as medians with Interquartile Range (IQR) and 95% Confidence Intervals (95% CI). Non-parametric tests (Mann–Whitney or Kruskal–Wallis) were used for comparisons between groups. For categorical data (e.g., pathogen), the absolute and relative frequencies (%) were used, and comparisons between groups were carried out using the chi-square test. For all tests, a *p* value ≤ 0.05 was considered significant, and all values presented are two-tailed. Statistical analyses were performed with IBM SPSS Statistics version 28 (IBM Corp., Armonk, NY, USA), and figures were created with GraphPad Prism software version 10 (GraphPad Software, Boston, MA, USA).

## 3. Results

### 3.1. Participant Characteristics

During the 16-year period from 1 January 2007 to 31 December 2022, a total of 2418 children (43.2% female) with culture-positive AOMO were examined at the ENT and Pediatric Outpatient Departments or the Emergency Department, and 2554 culture-positive MEF samples were obtained. More than one otopathogens were isolated in 202 samples (7.9%).

**Figure 1:**
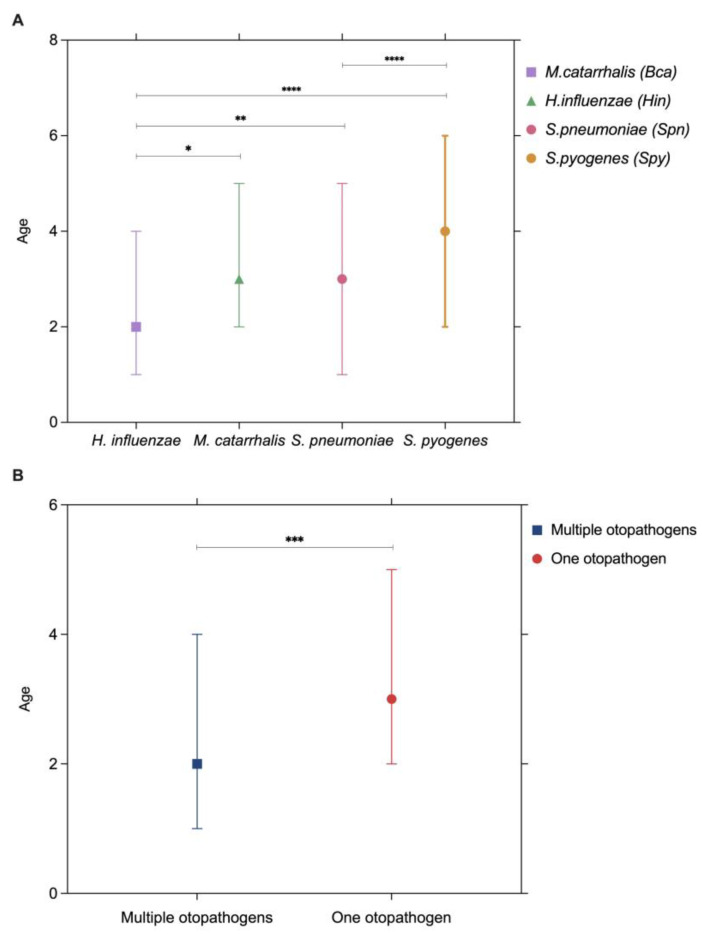
Median age (with interquartile range, IQR) of children with culture-positive AOMO during 2007–2022: (**A**) By otopathogen isolated (Kruskal–Wallis test and post hoc multiple comparisons) and (**B**) By number of otopathogens isolated (Mann–Whitney test). Statistical significance: * ≤0.05, ** ≤0.01, *** ≤0.001, **** ≤0.0001.

The age and gender of the children with AOMO included in our study—overall and by isolated pathogen or number of isolated pathogens—are depicted in Table 1. The median age of the children was 3 years (IQR: 1–5 years). A statistically significant difference in median age depending on the isolated pathogen was observed (Figure 1A). *H. influenzae* was isolated more frequently in younger children compared to the other pathogens (median age of 2 vs. 3 for *S. pneumoniae* and *M. catarrhalis*, and 4 for *S. pyogenes*, *p* < 0.05). *S. pyogenes* was more commonly isolated in older children compared to *S. pneumoniae* (median age 4 vs. 3, *p* < 0.001). Additionally, polymicrobial episodes (>1 otopathogen isolated) occurred more often in younger children (median age of 2 years vs. 3 years when one pathogen was isolated, *p* < 0.001, Figure 1B).

### 3.2. Comparison of Pathogen Frequency Among AOMO Cases Between the Two PCV Periods

**Figure 2:**
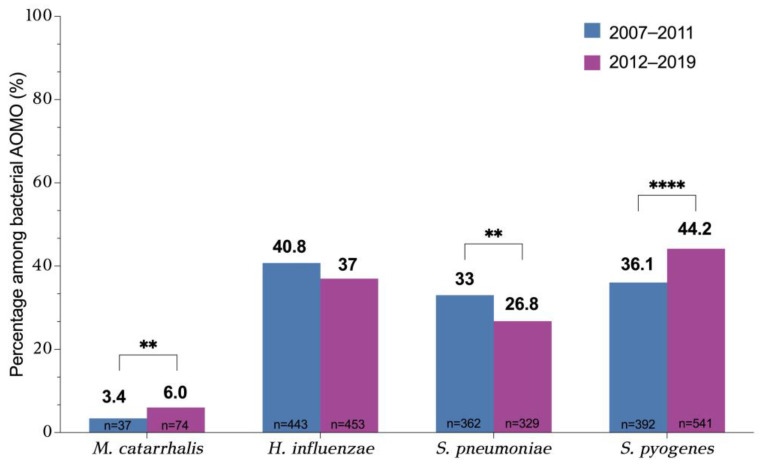
Percentages of each pathogen isolated from culture-positive AOMO cases during the PCV7-period (2007–2011) and the PCV13-period (2012–2019). Numbers inside the bars represent the number of isolates. Comparisons were made with the chi-square test. Statistical significance: ** ≤0.01, **** ≤0.0001.

When the two PCV periods were compared, a shift in the predominant pathogens was observed (Figure 2). During the PCV7-period, *H. influenzae* was the predominantly isolated otopathogen (40.8% of AOMO cases), followed by *S. pyogenes* (36.1%) and *S. pneumoniae* (33%). In the PCV13-period, the proportion of cases attributed to *S. pneumoniae* significantly decreased compared to the PCV7-period (from 33% to 26.8%, *p* = 0.0012). The proportion of *H. influenzae*-AOMO cases also decreased in the PCV13-period (to 37%), but the decrease was not statistically significant. In contrast, the proportion of *S. pyogenes* and *M. catarrhalis* cases increased significantly (to 44.2%, *p* < 0.0001 and 6%, *p* = 0.003, respectively), and *S. pyogenes* became the predominant pathogen during the PCV13-period.

### 3.3. Comparison of the Antimicrobial Susceptibility of Isolated Pathogens

**Figure 3:**
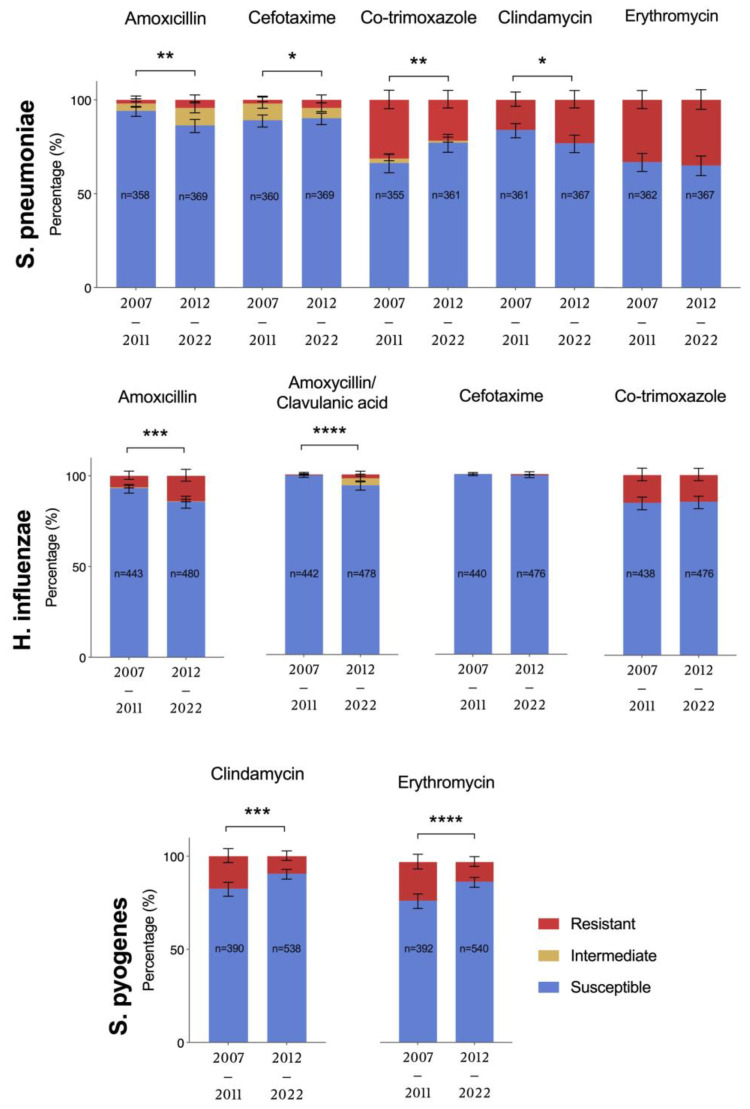
Susceptibility percentages of *S. pneumoniae*, *H. influenzae*, and *S. pyogenes* against various antimicrobial agents during the PCV7-period and PCV13-period. Numbers inside the bars represent the number of isolates tested for resistance against the specific antimicrobial. Error bars represent 95% confidence intervals for the proportion of resistant isolates in each period. Comparisons were made with the chi-square test. Statistical significance: * ≤0.05, ** ≤0.01, *** ≤0.001, **** ≤0.0001.

The susceptibility of the isolated pathogens against commonly used antimicrobials was tested and compared between the two PCV-periods (Figure 3). A significant increase in the percentage of pneumococcal strains resistant to clindamycin and cefotaxime was observed, from 16.1% to 22.8% (*p* = 0.039) and from 2% to 4,5% (*p* = 0.019), respectively. The percentage of erythromycin-resistant strains remained stable (33.9% vs. 33.7%, *p* = 1). Regarding pneumococcal susceptibility to amoxicillin, an increase of “susceptible-increased exposure” strains was observed (from 4.4 to 10.4%, *p* = 0.003), while the percentage of resistant strains remained very low (from 0% to 0.7%). Interestingly, the resistance of *S. pneumoniae* against co-trimoxazole significantly decreased from 31.2% to 22.4% (*p* = 0.012). Regarding *H. influenzae*, the proportion of strains resistant to ampicillin increased from 6.3% to 13.9% (*p* < 0.001), and resistance to amoxycillin–clavulanic acid increased from 0.5% to 2.1% (*p* < 0.0001). However, the susceptibility patterns to third-generation cephalosporins and co-trimoxazole remained stable (<0.7% and ~15%, respectively). All *H. influenzae* isolates were categorized as “susceptible-increased exposure” to oral cefuroxime. A notable significant reduction of erythromycin- and clindamycin-resistant *S. pyogenes* strains was observed, from 21.4% to 10.8% (*p* < 0.001) and from 17.4% to 9.3% (*p* = 0.001), respectively. Finally, the percentage of resistant *M. catarrhalis* strains against amoxicillin-clavulanic acid, cefotaxime, erythromycin, and co-trimoxazole remained low (≤3.1%) across both PCV periods.

### 3.4. Pneumococcal Serotypes

**Figure 4:**
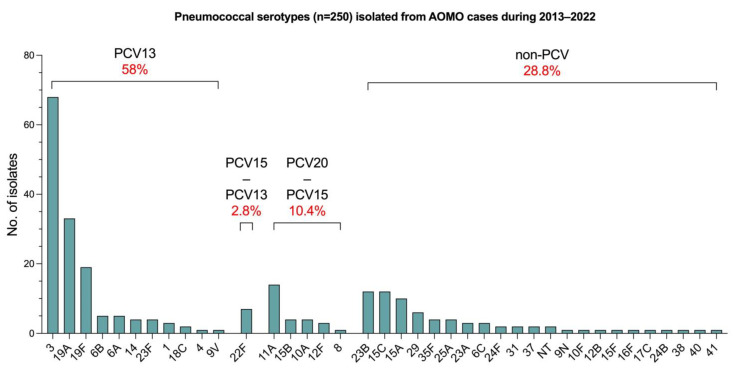
Distribution of *S. pneumoniae* serotypes among serotyped pneumococcal isolates from bacterial AOMO cases during the years 2013–2022, and coverage by the novel higher-valency PCVs (brackets).

Serotyping was available for 250 pneumococcal isolates during 2013–2022. A total of 38 serotypes were identified. Among serotyped strains, almost half (48%) belonged to three serotypes: serotype 3 was the most frequently identified isolate (27.2%), followed by 19A (13.2%) and 19F (7.6%). Other identified serotypes included mainly non-PCV13 vaccine serotypes, such as 11A (5.6%), 23B (4.8%), 15C (4.8%), 15A (4%), 22F (2.8%), 29 (2.4%), 6A (2%), and 6B (2%). The remaining 23.6% included serotypes 1, 4, 6C, 8, 9N, 9V, 10A, 10F, 12B, 12F, 14, 15B, 15F, 16F, 17C, 18C, 23A, 23F, 24B, 24F, 25A, 31, 35F, 37, 38, 40, 41, and 2 non-typeable isolates, each representing less than 2% of the isolates available for serotyping. No outstanding trend in the annual serotype distribution was observed regarding any of the identified serotypes. It is noteworthy that serotypes included in PCV13 were overall responsible for 58% of the AOMO cases for which serotyping was available (Figure 4). Although non-PCV13 serotypes were present throughout the study period and accounted for 27% to 59% of serotyped isolates annually, no consistent increase over time was observed. The yearly distribution fluctuated, and the limited number of isolates available for serotyping each year may have hindered the identification of subtle trends in serotype emergence. The recently available 15-valent PCV and 20-valent PCV vaccines include additional serotypes that were identified in 2.8% and 10.4% of the serotyped pneumococcal-AOMO samples, respectively. Finally, 28.8% of the identified serotypes are not currently included in any licensed PCV.

Susceptibility profiles against amoxicillin and cefotaxime for the three most frequently detected serotypes (among those serotyped)—3, 19A, and 19F—were compared to the overall distribution observed among all S. pneumoniae isolates collected from 2012 to 2022. Statistically significant differences were observed for both antimicrobials (Supplementary Figure S1). Serotype 3 isolates had a significantly lower proportion of amoxicillin-resistant strains compared to the total pneumococcal population [0% (95% CI: 0–5.8%) vs. 4.3% (95% CI: 2.7–6.9%,) *p* = 0.02], as well as compared to serotypes 19A and 19F [13% (95% CI: 4.5–32.1%), *p* < 0.0001, and 12.5% (95% CI: 2.2–36%), *p* = 0.001]. In contrast, serotype 19A showed a significantly higher resistance rate to amoxicillin compared to both the overall population (*p* = 0.0002).

With respect to cefotaxime, both 19A and 19F exhibited significantly higher resistance rates compared to the overall pneumococcal population [19A: 8.3% (95% CI: 1.5–25.9%) vs. 66.7% (95% CI: 46.7–82%%), *p* = 0.001; 19F: 20.8% (95% CI: 9.3–40.5%), *p* = 0.002], and to serotype 3 [4.2% (95% CI: 0.2–20.2%), *p* = 0.003 vs. 19A; *p* = 0.001 vs. 19F). In contrast, the resistance profile of serotype 3 to cefotaxime was similar to that of the overall pneumococcal population.

## 4. Discussion

In this hospital-based retrospective study, the effect of the switch from PCV7 to PCV13 on the microbiology of AOMO was examined. The results of our study are in line with the global trend toward the replacement of pneumococcus as the predominant pathogen involved in bacterial AOM, as recorded in the literature. In a recent study by Levy et al., the most frequently isolated otopathogen after the introduction of PCV13 was *H. influenzae* (48.4% of bacterial AOM), followed by *S. pyogenes* (34.7%) and *S. pneumoniae* (27.8%) [22]. Pichichero et al. reported *H. influenzae* and *S. pneumoniae* as the predominant pathogens in bacterial AOM cases after the introduction of PCV7, each representing 31% of the isolated bacteria [23]. After the switch to PCV13, a further decrease in the isolation rate of *S. pneumoniae* among MEF cultures to 24% was observed. Our data show a replacement of pneumococcus by *S. pyogenes* and *H. influenzae* as the predominant bacteria involved in AOMO. Overall, the most frequently isolated pathogen in our study was *S. pyogenes*. This was anticipated as this pathogen is most frequently associated with AOM complicated by spontaneous otorrhea [24,25].

The introduction of PCV13 has been shown to reduce not only pneumococcal AOM but also AOM caused by other pathogens such as *H. influenzae*. This may reflect an indirect effect of PCV13. By preventing early AOM episodes caused by *S. pneumoniae*, the vaccine may reduce the risk of progression to recurrent or more complex forms of AOM, which often involve non-pneumococcal pathogens such as *H. influenzae*. Ben-Shimol et al. [17] reported a marked decline not only in pneumococcal AOM but also in nontypeable *H. influenzae* and mixed infections following the sequential introduction of PCV7 and PCV13, supporting this hypothesis. Similarly, large-scale studies from the U.S. and Turkey documented significant reductions in overall AOM incidence after PCV13 replaced PCV7 [26,27].

The antibiotic resistance of otopathogens during the two PCV-periods was also examined. Pneumococcal resistance to amoxicillin, cephalosporins, and clindamycin increased in the PCV13-period, while resistance to erythromycin remained stable after the significant reduction recorded in previous years [21]. Of note, a significant decrease in pneumococcal resistance to cotrimoxazole was recorded. It appears that the switch from PCV7 to PCV13 failed to further reduce the pneumococcal resistance to common antibiotics, with the exception of cotrimoxazole. In contrast, a systematic review and meta-analysis of the effect of PCV on antibiotic resistance published in 2021 reported a significant reduction in pneumococcal resistance to penicillin, cephalosporins, cotrimoxazole, and macrolides [28]. However, the resistance rates recorded during the PCV13-period in the present study are similar to the current global resistance rates reported in the literature [29]. Greece has been struggling with antibiotic overuse, especially during the previous decades. Although vaccines are an important weapon in the fight against antibiotic resistance, they are not sufficient unless accompanied by the rational use of antimicrobials. Moreover, a significant burden of the disease is attributed to PCV13-included pneumococcal serotypes 3, 19A, and 19F, together accounted for almost half of the pneumococcal AOMO cases for which serotyping was available during the PCV13-period. This is in line with previous studies recording a high prevalence of these serotypes among AOM cases despite widespread vaccination with PCV13 [30]. As higher valency PCVs are now becoming available in many countries (i.e., 15-valent PCV, 20-valent PCV), it remains to be seen whether these formulations will be able to further reduce pneumococcal AOMO by reducing the circulation of additional serotypes and whether they will be effective against persistent PCV13-included serotypes.

On the contrary, the large reduction in resistance of *S. pyogenes* to erythromycin—though probably unrelated to pneumococcal vaccination—is particularly encouraging and highlights a significant improvement in the rational use of macrolides. The decrease in *S. pyogenes* resistance to both erythromycin and clindamycin is in contrast with the global increase recorded by the CDC from 2006 until 2022 [31]. National guidelines for the treatment of several infections, including AOM, were published for the first time in Greece in 2007 [32] and appear to have played a key role in promoting more rational use of antibiotics. The 2007 guidelines included macrolides as an alternative option without specifying restrictions. In contrast, the updated 2015 guidelines emphasized more cautious use of macrolides, reserving them for patients with β-lactam allergies, and instead recommended penicillins, cephalosporins, or clindamycin as first-line antibiotics for infections often caused by *S. pyogenes* [32]. In addition, macrolides are not recommended as first-line treatment for community-acquired respiratory infections. Indeed, the use of macrolides and the circulation of certain *S. pyogenes* emm strains, which are associated with increased macrolide resistance, have decreased in recent years in Greece [33,34]. This is further supported by national surveillance data from the ESAC-Net study, which showed a marked decline in the consumption of macrolides, lincosamides, and streptogramins in Greece between 1997 and 2017 [35]. In line with the observed decrease in resistance of *S. pyogenes* to macrolides and clindamycin in this study are recent data from the same region where the low resistance rates of a large collection of clinical *S. pyogenes* strains are sustained [36].

In the present study, we observed statistically significant differences in the median age depending on the bacterial cause of AOMO. Younger children were more frequently affected by *H. influenzae* AOMO (median age: 2 years) and older children by *S. pyogenes* (median age: 4 years). Moreover, multiple otopathogens were more likely to be isolated from younger children (median age: 2 years). According to the literature, the peak incidence of AOM is between 6 months and 2 years of age. The older median age observed in our study (3 years of age) is probably due to the fact that the MEF samples represent cases with otorrhea, which are known to be present in slightly older children compared to uncomplicated AOM [25]. Moreover, *S. pyogenes* is a pathogen isolated more frequently in older children [25,37]. Thus, the older median age of children presenting with otorrhea in this study may have contributed to the high percentage of *S. pyogenes* detection.

Regarding the distribution of pneumococcal serotypes in our study, our findings align with recent European data, which also highlight serotypes 3 and 19A as among the most prevalent causes of pneumococcal disease despite PCV13 inclusion. According to the 2022 ECDC report on invasive pneumococcal disease (IPD), the most common serotypes in Europe were 3, 8, 19A, 22F, 6C, 23B, 9N, 4, 23A, 11A, and 15A, accounting for nearly 74% of all typed isolates [38]. In our cohort, serotype 3 was also the most frequently identified strain, followed by 19A and 19F. Several non-PCV13 serotypes commonly seen in IPD across Europe—such as 11A, 23B, 15A, and 22F—were also observed in our study. These findings underscore the persistence of certain serotypes despite high PCV13 coverage and point to ongoing shifts in serotype distribution that may influence future vaccine strategies. Notably, serotype 3 persists despite PCV vaccination in many countries [39,40,41,42]. This persistence has been linked to unique biological characteristics—including continuous synthase-mediated capsule production, excessive release of capsular polysaccharide (CPS), and a non-covalently attached capsule—that hinder effective antibody-mediated clearance. These mechanisms, which may explain the limited vaccine effectiveness against serotype 3, have been recently reviewed in detail by Luck et al. [43]. Interestingly, despite its continued prevalence, serotype 3 showed significantly lower resistance rates compared to the total pneumococcal population. In contrast, serotype 19A—well recognized for its link to antimicrobial resistance—exhibited notably higher resistance levels. These observations align with the existing literature on the reduced β-lactam susceptibility of serotype 19A [44,45,46]. Nonetheless, the limited number of serotyped isolates constrains our ability to assess potential associations between resistance trends and shifts in serotype distribution. Further research with larger datasets is warranted to clarify these patterns and their clinical relevance.

Despite the above important findings, the present study is subject to several limitations. First, this is an observational study, and the results warrant careful interpretation. Observational studies are useful in revealing correlations and possible causal relationships, but they are not generally suitable to support causality with high certainty. However, they are useful when interventional studies are hard to implement, as is the case for the effect of PCV13 on AOMO due to the already wide implementation of PCV7 and the introduction of both of these vaccines in the NIP. While the study period spans both the use of PCV7 and its subsequent replacement by PCV13 in the national immunization program, individual vaccination data for participants were not available. Therefore, observed changes in microbial patterns over time are interpreted in the context of broader vaccine implementation trends, rather than direct individual-level vaccine impact. Another limitation of this study is that it included only children with AOMO who sought medical care in a tertiary hospital, possibly underestimating the frequency of AOMO in the population because cases with spontaneous resolution or successful treatment in a primary healthcare facility were not included. It should be noted that no central (national or regional) medical registry existed during the years of this study that would make it possible to study the true incidence of AOMO in the population. Furthermore, these population groups are under-vaccinated compared to the general population in Greece [47,48]. According to the available data, PCV vaccine uptake in Greece was already high prior to PCV13’s introduction. A 2009–2011 study at a large pediatric outpatient clinic in Athens found that 79% of children ≤ 2 years had received at least two doses [49]. In 2013, a national survey of preschoolers in nurseries and kindergartens reported that 82.3% had received three doses by 12 months, despite frequent delays in booster administration [50]. Most recently, a 2024 report analyzing anonymized data from the Electronic Prescription System and the Social Security Registration Number Registry estimated that ≥90% of children born between 2013 and 2022 received at least two PCV doses by 24 months [51]. Therefore, it is possible that the impact of pneumococcal vaccination on the epidemiology of the disease in the general population may be even greater than has been shown in this study. Finally, only 250 of the 644 *S. pneumoniae* isolates were serotyped, possibly limiting the representativeness of this subset regarding serotype distribution. However, these isolates were randomly selected across the study period, with no restrictions based on patient demographics or clinical settings, suggesting that the serotyped subset is likely representative of the broader *S. pneumoniae* population.

## 5. Conclusions

In the years following the shift of vaccination from PCV7 to PCV13, a significant decrease in pneumococcal and *H. influenzae*-related AOMO was recorded. The predominant bacterial causes of AOMO in our area are now *S. pyogenes* and *H. influenzae*. Pneumococcus, however, remains an important bacterial cause of AOMO, associated with high rates of macrolide resistance. Among the *S. pneumoniae* isolates available for serotyping during the PCV13-period, serotype 3 was the most frequently detected, followed by 19A. A significant decrease in the resistance rate of *S. pyogenes* to clindamycin and macrolides was recorded. This highlights the need to combine the benefits of vaccination with rational antibiotic use. Evidence-based guidelines can and should play a decisive role in this respect. According to our findings, amoxicillin remains an effective empirical treatment for pediatric AOMO. The results of the present study can contribute to an evidence-based approach for the empirical treatment of AOMO based on the regional resistance rates. However, as our study is based on cases presented to a tertiary care center, the findings may not fully capture the spectrum of pathogen circulation in the broader community, particularly among children managed in primary care settings or not seeking medical attention. This highlights the need for wider epidemiological surveillance to inform vaccination strategies and antibiotic stewardship in real-world conditions.

## 6. Take-Home Messages

- Serotype replacement is an ongoing phenomenon in pneumococcal circulation, even in communities with high vaccine coverage, and influences the etiology of common pediatric infections such as AOMO.
- Following widespread PCV13 use, *H. influenzae* and *S. pyogenes* have emerged as leading causes of AOM with otorrhea, underlining the shifting etiology of pediatric ear infections.
- Serotype 3 remains a dominant pneumococcal strain despite PCV13 coverage, highlighting the need for next-generation vaccines or improved immunogenicity against this serotype.
- Coordinated strategies that combine vaccination, surveillance, and antibiotic stewardship are essential to maintain gains in disease prevention and to control antimicrobial resistance.

## Figures and Tables

**Table 1:** Demographic characteristics (age and gender) of children with culture-positive AOMO according to the isolated pathogen and the number of pathogens isolated.

	Age, Median (IQR)	*p*-Value	Females, N (%)	*p*-Value
*S. pneumoniae*	3 (1–5)	<0.001 ^	261 (43.4)	0.54 †
*H. influenzae*	2 (1–4)		308 (41.1)	
*S. pyogenes*	4 (2–6)		402 (44.3)	
*M. catarrhalis*	3 (2–5)		24 (39.3)	
*Single-pathogen episodes*	3 (2–5)	0.001 ^^	996 (42.9)	0.476 †
*Polymicrobial episodes (>1 pathogen)*	2 (1–4)		88 (45.6)	
Total culture-positive AOMO cases	3 (1–5)		1084 (43.2)	

^ Kruskal–Wallis, ^^ Mann–Whitney, † chi-square.

## Data Availability

The data presented in this study are not publicly available due to legal issues (hospital records) but are available on request from the corresponding author.

## Supplementary Materials

The following supporting information can be downloaded at: https://www.mdpi.com/article/10.3390/pathogens14050494/s1, Figure S1: Susceptibility percentages of total S. pneumoniae isolates from 2012–2022 and pneumococcal serotypes 3, 19A, and 19F isolates among serotyped strains from 2013–2022 against amoxicillin (A) and cefotaxime (B).

## Abbreviations

The following abbreviations are used in this manuscript:

AOM	Acute otitis media
AOMO	Acute otitis media with otorrhea
COVID-19	Coronavirus disease 2019
ENT	Ear–nose–throat
MEF	Middle ear fluid
PCV	Pneumococcal conjugate vaccine
PCV7	7-valent pneumococcal conjugate vaccine
PCV13	13-valent pneumococcal conjugate vaccine
AOM	Acute otitis media
