# Supplementary material for: Bacterial Acute Otitis Media Complicated with Otorrhea in a Children’s Hospital in the Era of Pneumococcal Conjugate Vaccines
Source: Pathogens. 2025 May 17;14(5):494. doi: 10.3390/pathogens14050494 (PMC12114500; doi:10.3390/pathogens14050494)
Supplement: Supplementary file 1 [file pathogens-14-00494-s001.zip › pathogens-3587922-supplementary.pdf]

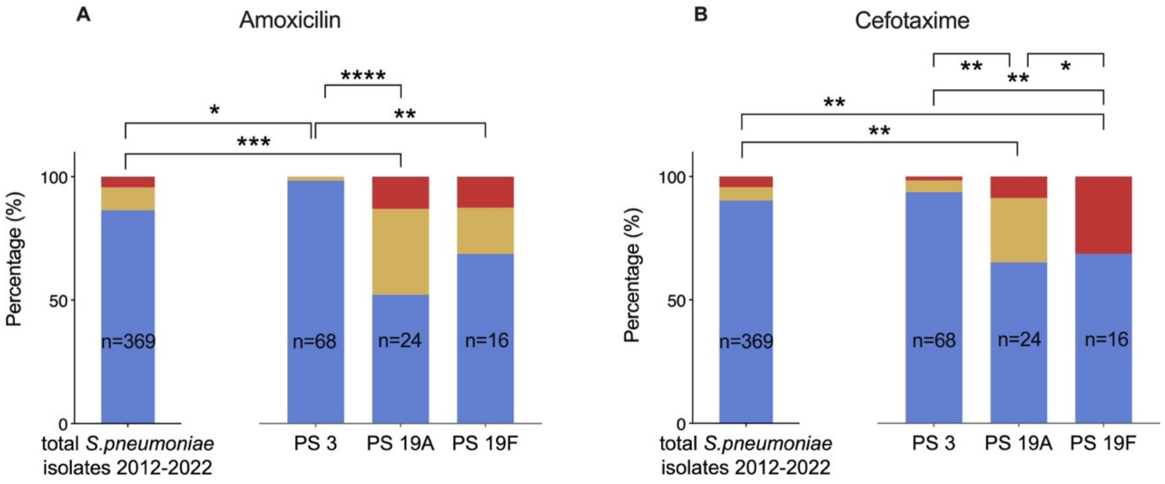

Figure S1. *Susceptibility percentages of total S. pneumoniae isolates from 2012-2022 and pneumococcal serotypes 3, 19A, and 19F isolates among serotyped strains from 2013-2022 against amoxicillin (A) and cefotaxime (B). The numbers inside the bars represent the number of isolates tested for resistance against the specific antimicrobial. Error bars represent 95% confidence intervals for the proportion of resistant isolates in each period. Comparisons were made with the chi-square test. Statistical significance: * ≤0.05, ** ≤0.01, ***≤0.001, ****≤0.0001.*
